# Mechanical Properties and Microscopic Mechanism of Shield Tunnel Spoil Stabilized with ESCA

**DOI:** 10.3390/ma19112345

**Published:** 2026-06-01

**Authors:** Liandi Zhao, Henggen Zhang, Xiaoge Yu, Xujiayin Zhao, Jinwen Chen

**Affiliations:** 1 Shandong Key Laboratory of Technologies and Systems for Intelligent Construction Equipment, Shandong Jiaotong University, No. 5001 Haitang Road, Changqing District, Jinan 250357, China; 504009@sdjtu.edu.cn (L.Z.);; 2 School of Transportation and Civil Engineering, Shandong Jiaotong University, Jinan 250357, China; 3 Shandong Engineering Research Center of Marine Exploration and Conservation, Ocean University of China, Qingdao 266100, China; 4 Shandong High-Speed Maintenance Industry Co., Ltd., Jinan 250014, China

**Keywords:** shield tunnel spoil, early-strength cementitious agent, solidification, mechanical properties, microstructure, computed tomography

## Abstract

The efficient treatment and resource utilization of shield tunnel spoil (STS) are important for sustainable underground construction in China. To improve the early mechanical performance and microstructural compactness of stabilized STS, this study investigated the solidification effect of a novel early-strength cementitious agent (ESCA) and compared it with ordinary Portland cement (P.O 42.5). Macroscopic mechanical tests, including unconfined compressive strength (UCS), stress–strain behavior, mass, and P-wave velocity measurements, were combined with scanning electron microscopy (SEM) and computed tomography (CT) analyses to reveal the mechanical response and microstructural mechanisms of stabilized STS. The results indicate that, compared with P.O 42.5, ESCA exhibits superior fluidity at lower water-to-solid (w/s) ratios, significantly shorter setting times, and higher compressive strength at all curing ages. The solidification efficiency of ESCA for STS is notably superior to that of P.O 42.5, with the peak strength, elastic modulus, mass, and P-wave velocity of ESCA-solidified specimens being higher than those of P.O 42.5-solidified specimens across the five dosages. Furthermore, ESCA material bonds more tightly with STS particles, resulting in lower porosity and a denser microstructure under the same stabilizer dosage. Overall, the combination of macroscopic mechanical properties and microstructural characterization demonstrates that ESCA material exhibits significant advantages in the efficient solidification and resource utilization of shield tunnel spoil.

## 1. Introduction

With the rapid development of urban rail transit, tunnel engineering, and underground space development in China, shield construction technology has been widely applied. Consequently, a large amount of STS has been generated, which has become an urgent challenge in engineering construction [1]. STS typically exhibits characteristics such as high water content, uneven particle size distribution, low strength, and poor bearing capacity, classifying it as a typical engineering waste soft soil [2]. If not disposed of properly, it not only occupies substantial land resources but may also lead to secondary problems such as environmental pollution and soil erosion [3]. Achieving efficient treatment and resource utilization of STS is not only a practical requirement for engineering construction but also an important approach to promoting green construction and sustainable development.

Regarding the disposal methods of STS, traditional approaches mainly include direct stacking, landfilling, and simple backfilling [4]. These conventional disposal methods generally suffer from drawbacks such as large land occupation, high environmental risks, and low resource utilization efficiency [5]. With the continuous increase in environmental protection requirements, the application of solidification/stabilization technology to improve the engineering properties of spoil and achieve its resource utilization has become a mainstream research direction in this field [6,7]. Due to its excellent cementitious properties, cement has been widely used, and numerous scholars have conducted systematic studies on the mechanisms and effects of cement stabilization of soils. For instance, Alazigha et al. [8] systematically elucidated the stabilization mechanism of cement on expansive soils, pointing out that the hydration products of cement clinker with water, namely calcium silicate hydrate (C-S-H) gel and calcium hydroxide (CH), form a spatial network structure within the soil, encapsulating and cementing soil particles, thereby transforming the loose soil into an integrated structure. Yang et al. [9] investigated the mechanical properties of silty clay stabilized by cement combined with municipal solid waste incinerator bottom ash, exploring the effects of bottom ash content and curing temperature on the mechanical properties and microstructure of the cement-stabilized silty clay. Ahmad et al. [10] employed cement, cement kiln dust, and limestone powder for the stabilization of sandy soil contaminated with diesel and crude oil, finding that the combined use of these materials significantly enhanced the compaction characteristics, unconfined compressive strength, permeability resistance, and leaching resistance of the contaminated soil. Liu et al. [11] investigated the effect of cement stabilization on the soil-water characteristic curve and deformation behavior of soft clay and found that with the increase in cement content, the water retention capacity of the soil was significantly enhanced, the hysteresis effect of the soil-water characteristic curve became more pronounced, and the deformation resistance of the soil was effectively improved. The aforementioned studies collectively indicate that the mechanical properties of stabilized soil are influenced by multiple factors, with the stabilizer content being one of the key controlling factors [12]. Nevertheless, traditional cement-based stabilization methods still exhibit several inherent limitations: firstly, cement production is characterized by high energy consumption and significant carbon emissions, which is inconsistent with the development trend of green building materials [13]; secondly, for STS, which is typically characterized by complex compositions and a high content of fine particles, the stabilization efficiency of cement is limited [14]; thirdly, the stabilized soil often develops strength slowly at early ages, making it prone to cracking or shrinkage deformation during construction, thereby compromising the safety of engineering structures [15].

To reduce cement consumption, in recent years, the academic community has actively explored the partial or complete replacement of cement with industrial solid wastes such as ground granulated blast furnace slag (GGBS), fly ash (FA), and flue gas desulfurization gypsum (FGDG), aiming to enhance the environmental friendliness and economic viability of stabilization systems [16,17]. GGBS, a silica-alumina-rich amorphous glassy material produced during ironmaking, exhibits significant pozzolanic activity and serves as a high-quality cementitious substitute, contributing to improved long-term strength and durability of stabilized soils [18]. FA, a fine fly ash collected from coal-fired power plants, possesses spherical particle morphology that exerts a “ball-bearing effect,” improving the fluidity of mixtures, reducing water demand, and optimizing the workability of stabilized soils [19]. FGDG, a byproduct of the flue gas desulfurization process, promotes the formation of cementitious products such as calcium aluminate hydrate and calcium silicate hydrate upon incorporation into stabilized soils, enhancing interparticle bonding while regulating the setting characteristics and volume expansion behavior of the stabilization system [20]. Previous studies have investigated the effectiveness of different industrial solid wastes in cement-based soil stabilization systems [21]. Shun et al. [22] developed a composite stabilizer suitable for the solidification of dredged river sludge using industrial solid wastes such as carbide slag, GGBS, and FA as primary raw materials, supplemented with a small amount of cement, achieving satisfactory stabilization performance through optimized proportioning. Shen et al. [23] elucidated the stabilization mechanism of a stabilizer composed of GGBS, quicklime, sodium silicate, and gypsum powder on saline dredged soil, demonstrating that this stabilizer significantly enhances the unconfined compressive strength of the soil, forms a dense microstructure with reduced porosity, and effectively decreases soluble salt content, thereby substantially improving its engineering properties. Wang et al. [24] prepared an environmentally friendly material using GGBS and FGDG and systematically investigated its stabilization performance and mechanism on copper-contaminated soil. The results revealed that the abundant Ca^2+^ and SO_4_^2−^ in FGDG promote cementitious reactions, thereby enhancing the mechanical properties of the stabilized soil. Collectively, these studies demonstrate that incorporating industrial solid wastes into cement-based stabilization systems not only effectively reduces cement consumption but also exhibits promising potential for enhancing soil strength and stability, providing significant support for the development of green civil engineering materials [25,26].

In this study, the STS from the Longwan Yangtze River Tunnel in Wuhu was stabilized using a novel early-strength ESCA material. By comparing the physical and mechanical properties of STS stabilized with ESCA and P.O 42.5 at different dosages, combined with microstructural characterization techniques such as SEM and industrial CT, the micro-mechanisms of ESCA-stabilized muddy soil were systematically elucidated. This study aims to provide theoretical insights and technical references for the resource-oriented engineering application of STS.

## 2. Materials and Procedures

### 2.1. STS

**Figure 1:**
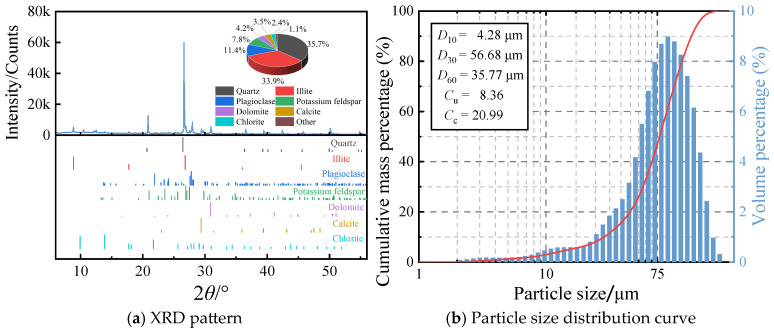
(**a**) XRD pattern and (**b**) particle size distribution curve of the STS.

The STS used in this experiment was sampled from the Longwan Yangtze River Tunnel in Wuhu City, Anhui Province. The tunnel project is located in the alluvial plain of the middle and lower reaches of the Yangtze River and was constructed using the slurry shield method. The stratigraphic structure at the construction site is relatively complex, with the upper part consisting of Quaternary loose sedimentary soil and the lower part composed of Cretaceous and Jurassic bedrock. The STS was collected from the lower part of the slurry settling tank at the construction site at a depth of 2.5 m. The obtained STS was first dried, and its mineral composition was analyzed using X-ray diffraction (XRD). The results, shown in Figure 1a, indicate that the STS is mainly composed of quartz (35.7%), illite (33.9%), plagioclase (11.4%), potassium feldspar (7.8%), dolomite (4.2%), calcite (3.5%), chlorite (2.4%), and other minerals (1.1%). The particle size distribution of the dried undisturbed STS was determined using a laser particle size analyzer, and the results are presented in Figure 1b. In its natural state, the STS appears black with a uniform particle distribution. According to the classification standard GB/T 50145-2007 [27], it is categorized as a coarse-grained STS. The basic physical properties of the STS were tested, and the results are summarized in Table 1.

### 2.2. ESCA

**Figure 2:**
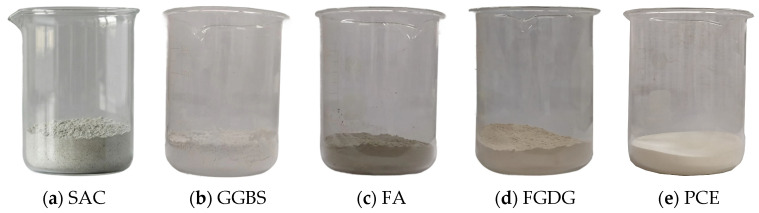
The components of ESCA.

**Figure 3:**
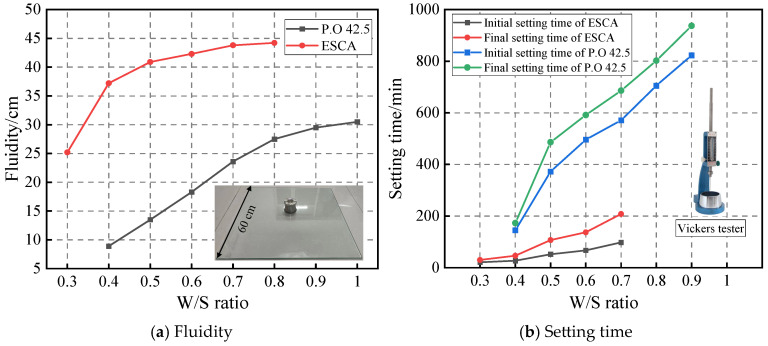
Comparison of (**a**) fluidity and (**b**) setting time between ESCA material and P.O 42.5 at different w/s ratios, showing the higher fluidity and shorter setting time of ESCA.

The ESCA used in this study is formulated by partially replacing sulphoaluminate cement (SAC), and its main components (Figure 2) include 45% SAC, 35% ground granulated blast furnace slag (GGBS), 12% fly ash (FA), 7% flue gas desulfurization gypsum (FGDG), and 1% polycarboxylate superplasticizer (PCE) water-reducing agent. It is characterized by a low carbon footprint, early strength, rapid setting, high strength, and slight volume expansion. To compare with the commonly used P.O 42.5, the fluidity of ESCA material and P.O 42.5 (both as cement pastes) was tested in the laboratory under different w/s ratios according to the specifications of GB/T 8077-2012 [28]. The results are shown in Figure 3a. Compared with P.O 42.5, ESCA exhibited high fluidity even at low w/s ratios, which is particularly advantageous for STS solidification. The paste with better fluidity can be mixed more uniformly with the STS, reducing the number of air bubbles in the solidified soil while enhancing its strength. Figure 3b presents the setting times of ESCA material and P.O 42.5 measured using a Vicat apparatus (Humboldt Mfg. Co., Ltd., Elgin, IL, USA). As shown in Figure 3b, due to the incorporation of sulphoaluminate cement and flue gas desulfurization gypsum, ESCA material exhibited shorter initial and final setting times than P.O 42.5 under the same w/s ratios, which is attributed to the rapid formation of ettringite (AFt). The extremely short initial and final setting times can shorten the construction period for solidifying STS with ESCA material, thereby improving engineering efficiency.

**Figure 4:**
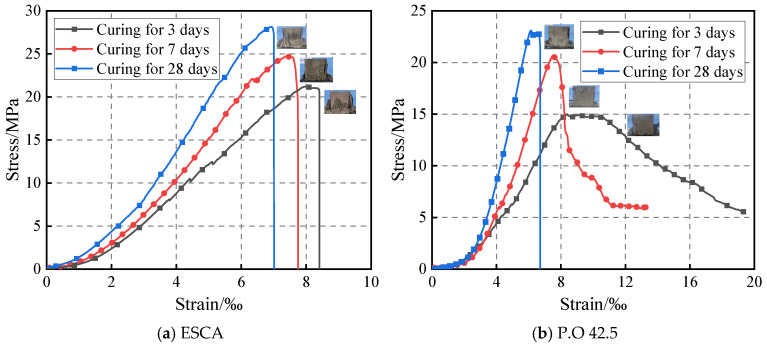
Strength curves of (**a**) ESCA and (**b**) P.O 42.5 at different curing ages, showing the higher compressive strength and more pronounced early-strength behavior of ESCA.

For each material and curing age, three cubic specimens with a side length of 70.7 mm were prepared at a w/s ratio of 0.4. The specimens were cured in a constant-temperature and humidity curing box at 20 ± 2 °C and a relative humidity of no less than 95% until the designed curing ages of 3, 7, and 28 days. The UCS of these specimens was then tested at the corresponding curing ages. Figure 3 presents the stress–strain curves of typical specimens obtained from the tests. As shown in Figure 4, the compressive strength of ESCA material was higher than that of P.O 42.5 at all three curing ages. At the curing age of 3 days, ESCA material already exhibited high strength and displayed strong brittle behavior under static loading. In contrast, P.O 42.5 specimens showed a plastic stage after peak stress and a relatively long compaction stage before peak stress at both 3 and 7 days of curing.

### 2.3. Experimental Procedure

**Figure 5:**
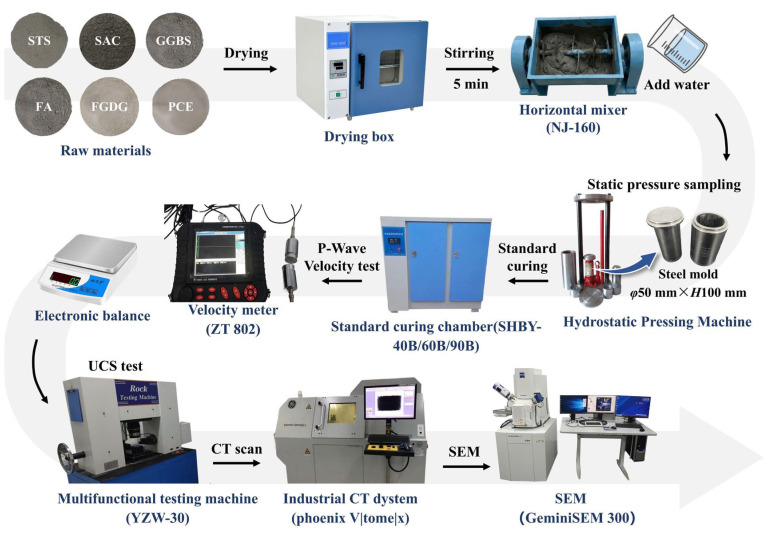
Experimental workflow and schematic diagram of the equipment.

After comparing the fundamental properties of ESCA and P.O 42.5, STS solidification tests were conducted using the two stabilizers at five dosages: 10%, 20%, 30%, 40%, and 50%. The stabilizer dosage was defined as the mass ratio of the stabilizer to the dry STS, i.e., *m*_s_/*m*_d_ × 100%, where *m*_s_ is the mass of the stabilizer, and *m*_d_ is the mass of the oven-dried STS. The STS was first oven-dried at 105 °C for 24 h. The dried STS and stabilizer were then weighed according to the designed proportions, mixed with water at 40% of the total dry mass of STS and stabilizer for 5 min, and compacted under constant pressure to prepare cylindrical specimens with dimensions of *φ*50 mm × 100 mm. For each stabilizer dosage and stabilizer type, three parallel cylindrical specimens were prepared and cured in a standard curing chamber at 20 ± 2 °C and a relative humidity of no less than 95% for 28 days. After curing, the specimens were first subjected to mass and P-wave velocity testing, followed by UCS tests. To analyze the solidification mechanisms of ESCA material and P.O 42.5 on STS from a microscopic perspective, both cured specimens and crushed fragments were used for industrial CT scanning and SEM analysis, respectively. UCS tests were conducted using a YZW-30 rock multifunctional testing machine (Jinan Puye Electromechanical Technology Co., Ltd., Jinan, China) at a displacement loading rate of 0.025 mm/min [29]. X-ray CT scanning was performed using a phoenix V|tome|x industrial CT system (Waygate Technologies, Wunstorf, Germany), yielding 1080 two-dimensional slices per specimen. These slices were utilized for three-dimensional (3D) reconstruction and the extraction of pores and cracks, with a spatial resolution of 93 μm during reconstruction. For SEM observation, crushed fragments collected after UCS testing were dried, mounted on conductive tape, and gold-coated prior to testing. SEM analysis was then carried out using a GeminiSEM 300 instrument (Carl Zeiss Microscopy GmbH, Oberkochen, Germany) to observe the morphology of hydration products and the bonding characteristics between particles. The detailed experimental procedure is illustrated in Figure 5.

## 3. Results and Discussion

### 3.1. Variation in P-Wave Velocity

**Figure 6:**
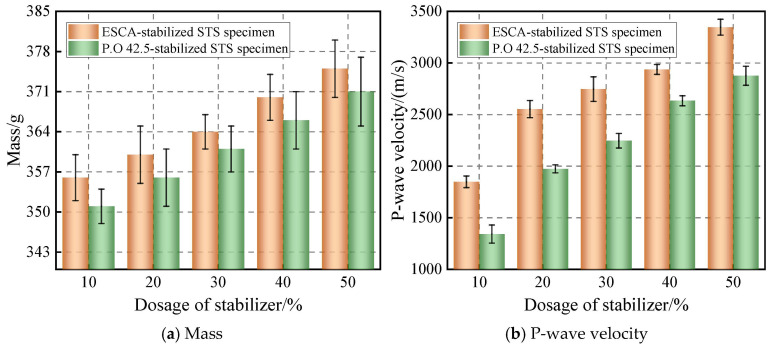
Variations in mass andP-wave velocity of STS specimens stabilized with ESCA and P.O 42.5 at different dosages, indicating the higher compactness of ESCA-stabilized specimens.

Figure 6 illustrates the variations in mass and P-wave velocity of stabilized STS specimens under different stabilizer dosages, with error bars representing the standard deviation of three parallel specimens. With increasing stabilizer dosage, the P-wave velocity of the specimens exhibited an upward trend, reflecting the positive regulatory effect of stabilizer dosage on the internal compact structure of the specimens. At stabilizer dosages of 10%, 20%, 30%, 40%, and 50%, the mass of ESCA-stabilized specimens was 4 g, 4 g, 3 g, 4 g, and 5 g higher than that of P.O 42.5-stabilized specimens, respectively, indicating that ESCA-stabilized specimens possessed slightly higher mass per unit volume under identical preparation conditions. This increase in mass is closely related to the superior fluidity of the ESCA paste and the resulting tighter particle packing. In terms of P-wave velocity response, ESCA-stabilized specimens exhibited higher P-wave velocities than P.O 42.5-stabilized specimens across all dosages, with values 1.38, 1.29, 1.22, 1.12, and 1.16 times those of the latter, respectively. This improvement can be attributed to the better fluidity of ESCA paste, which promotes more uniform coating of STS particles and tighter interparticle bonding. The resulting denser structure and reduced porosity provide a more continuous medium for elastic wave propagation, thereby increasing the P-wave velocity. In contrast, the P.O 42.5 slurry exhibits relatively poor fluidity, making it difficult to achieve the same degree of uniform mixing and dense packing under identical mixing conditions, resulting in a relatively higher internal porosity and a lower P-wave velocity response [30]. Collectively, the results from both mass and P-wave velocity measurements further substantiate the beneficial role of ESCA material in improving the structural compactness and uniformity of stabilized shield tunnel spoil.

### 3.2. Stress–Strain Curves

**Figure 7:**
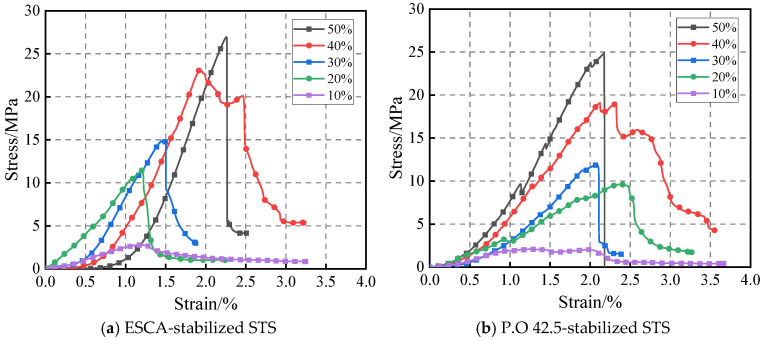
Stress–strain curves of (**a**) ESCA-stabilized STS and (**b**) P.O 42.5-stabilized STS at stabilizer dosages of 10%, 20%, 30%, 40%, and 50%.

After 28 days of curing, the stress–strain curves of STS specimens stabilized with ESCA and P.O 42.5 at different dosages are presented in Figure 7. The curves of both types of stabilized specimens exhibited typical stage-wise characteristics, sequentially undergoing the pore compaction stage, elastic deformation stage, yield failure stage, and post-peak softening stage. As the stabilizer dosage decreased, the post-peak curves of both stabilized specimens tended to become gentler, with the brittle failure characteristics gradually weakening and the plastic deformation characteristics becoming more pronounced, reflecting the modulating effect of stabilizer dosage on the failure mode of the specimens. Compared with ESCA-stabilized specimens, the pre-peak strain of P.O 42.5-stabilized specimens decreased significantly with decreasing stabilizer dosage, and the peak strength at each dosage was markedly lower than that of ESCA-stabilized specimens. This difference is mainly attributed to the excellent fluidity and early-age cementitious activity of ESCA material, which enables it to form more sufficient contact and bonding with STS particles even at lower dosages, thereby constructing a more continuous and dense skeleton structure, effectively enhancing the overall bearing capacity and deformation compatibility of the specimens. In contrast, at low dosages, P.O 42.5 provides less effective coating and bonding of fine particles, resulting in more weakly cemented zones and, consequently, lower strength and limited pre-peak strain.

### 3.3. Variation in Mechanical Parameters

**Figure 8:**
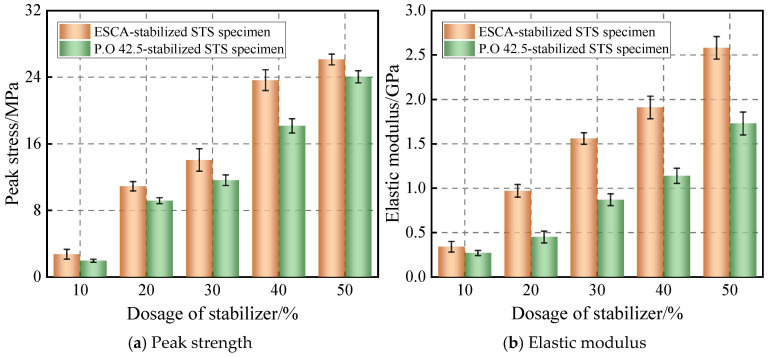
Variation in (**a**) peak strength and (**b**) elastic modulus of STS stabilized with ESCA and P.O 42.5, showing the superior strength enhancement and stiffness improvement of ESCA-stabilized specimens.

Based on the stress–strain curves shown in Figure 7, the mechanical parameters (including peak strength and elastic modulus) of the stabilized STS specimens were further calculated, and the results are presented in Figure 8. The elastic modulus was determined as the slope of the linear elastic segment of the curves in Figure 7. As shown in Figure 8, at stabilizer dosages of 10%, 20%, 30%, 40%, and 50%, the mean peak strength of ESCA-stabilized specimens was 1.40, 1.19, 1.21, 1.30, and 1.09 times that of P.O 42.5-stabilized specimens, respectively, while the mean elastic modulus was 1.26, 2.16, 1.79, 1.68, and 1.49 times that of the latter, respectively. This indicates that ESCA has a generally higher stabilization efficiency for STS than P.O 42.5, particularly at lower dosages. This enhanced performance is mainly attributed to the multi-component synergistic stabilization mechanism of the ESCA material. During the stabilization process, the SAC in ESCA undergoes rapid hydration, generating AFt and calcium aluminate hydrate (AFm), which contribute to early-age strength. Meanwhile, ground granulated blast furnace slag (GGBS) and fly ash (FA) are activated in the alkaline environment, undergoing pozzolanic reactions to form cementitious products such as C-S-H, which fill the interparticle pores. The abundant Ca^2+^ and SO_4_^2−^ provided by flue gas desulfurization gypsum (FGDG) further promote the formation and stabilization of AFt, enhancing the compactness of the cementitious system [31]. The interplay of these diverse hydration products collectively constructs a spatial network-like cementitious structure, thereby significantly improving the peak strength and elastic modulus of the stabilized soil. These findings are consistent with recent studies showing that industrial solid-waste-based composite stabilizers can improve the strength and stiffness of stabilized soils through synergistic hydration and pore-filling effects [32,33]. However, compared with conventional cement-based stabilization systems, ESCA exhibited a more pronounced strength enhancement at lower dosages, indicating its potential for reducing stabilizer consumption while maintaining satisfactory mechanical performance.

### 3.4. Failure Mode

**Figure 9:**
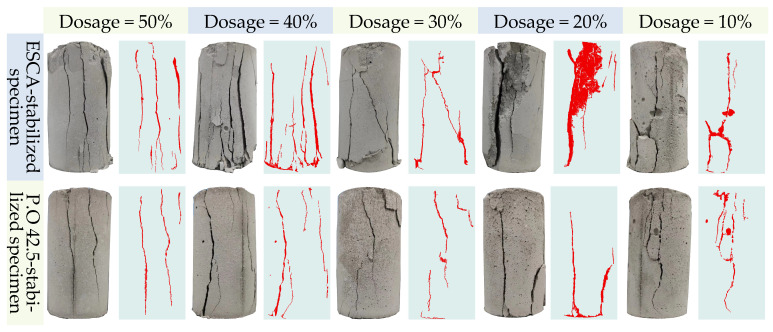
Failure modes of STS specimens stabilized with the two stabilizers at different dosages.

Figure 9 presents the typical failure mode of stabilized STS specimens after UCS testing. For clearer visualization of the crack development characteristics, the cracks in the failed specimens were extracted and marked in red. As shown in the figure, the stabilizer dosage exerts a significant regulating effect on the failure mode of the specimens. As the stabilizer dosage decreased, the crack propagation on the surface of P.O 42.5-stabilized specimens tended to become more complex, with a reduction in split cracks penetrating the specimen and an increase in shear cracks, reflecting a transition from brittle failure to shear failure as the degree of cementation weakened. For ESCA-stabilized specimens, when the stabilizer dosage was below 30%, the number of surface cracks decreased, while the spalling area increased, indicating a stronger local crushing characteristic during the failure process. Compared with P.O 42.5-stabilized specimens, ESCA-stabilized specimens exhibited more developed surface cracks at the same dosage, with more load-bearing units participating in failure. This difference is mainly attributed to the formation of a more continuous and high-strength cementitious network in the ESCA stabilization system at the microscopic scale. The rapid hydration reaction of SAC in the ESCA material generates a large number of needle-like crystals, which synergistically interact with the gel products formed by GGBS and FA in the alkaline environment, constructing a spatial skeleton structure with high cementation strength. This structure enables efficient stress transfer and distribution under loading, allowing more particles to participate synergistically in load-bearing. However, once the bearing capacity limit is reached, stress rapidly propagates through the dense cementitious network, leading to sudden brittle failure, which macroscopically manifests as dense cracking and significant spalling [34].

### 3.5. SEM Analysis

**Figure 10:**
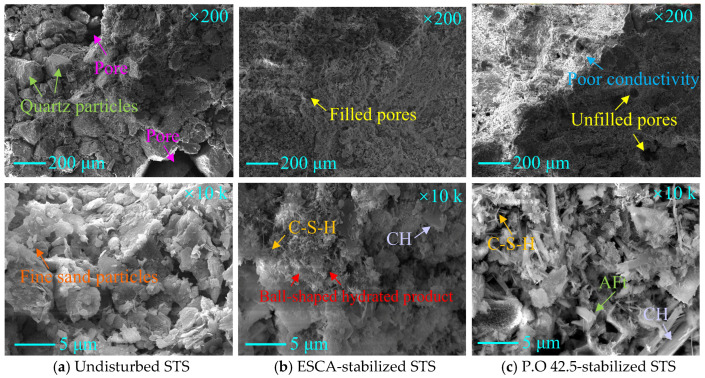
SEM images of undisturbed STS and STS specimens stabilized with ESCA and P.O 42.5, showing denser hydration products and tighter particle bonding in the ESCA-stabilized specimen. (**a**) Undisturbed STS; (**b**) ESCA-stabilized STS; (**c**) P.O 42.5-stabilized STS.

**Figure 11:**
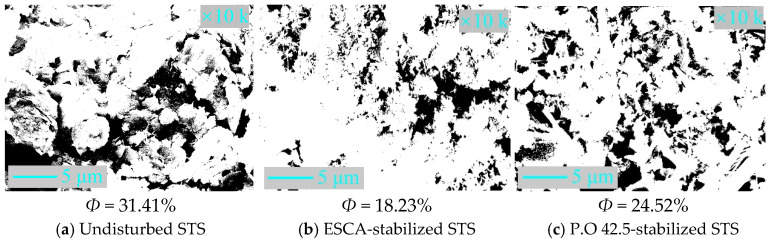
Pore distribution characteristics on the specimen surface obtained from binarized SEM images.

To reveal the stabilization mechanism of ESCA and P.O 42.5 on STS at the microscopic level, fragments of STS specimens stabilized with the two stabilizers at a dosage of 20% were collected after UCS testing for SEM observation. The results are presented in Figure 10, which also includes the microstructure of the original dried STS. Subsequently, binarization processing was conducted on the images with a magnification of 10,000× in Figure 10 to extract the surface pore features and calculate the pore area ratio (*Φ*), with the results shown in Figure 11. SEM observations indicated that the original STS contained a significant number of coarse mineral particles such as quartz, which were encased by weathered secondary minerals, exhibiting loose interparticle contact and a relatively porous structure. After stabilization with the two materials, at a magnification of 200×, it was evident that the hydration products in the ESCA-stabilized specimen exhibited a more pronounced cementing effect on the particles, resulting in tighter interparticle bonding and a denser surface pore structure compared to the P.O 42.5-stabilized specimen. Moreover, the P.O 42.5-stabilized specimen showed poor electrical conductivity during SEM imaging, with noticeable electron accumulation observed within the 200× field of view, reflecting uneven distribution of hydration products and poor continuity of the cementitious structure. At a magnification of 10,000×, abundant flocculent hydration products, along with needle-like C-S-H gels and plate-like CH crystals, were observed on the surface of the ESCA-stabilized specimen. According to the study by Zhu et al. [35], the flocculent hydration products are primarily composed of AFt, C-S-H, and calcium aluminum silicate hydrate (C-A-S-H) gels, with AFt crystals forming the skeletal framework and other gel products attaching to their surfaces to form a composite cementitious structure. In contrast, although hydration products such as AFt, CH, and C-S-H were also observed on the surface of the P.O 42.5-stabilized specimen, they were more sparsely distributed, with poor continuity of the cementitious network and significantly weaker pore-filling capability compared to the ESCA-stabilized specimen. The surface pore area ratio of the ESCA-stabilized specimen was 18.23%, while that of the P.O 42.5-stabilized specimen was 24.52%, representing a reduction of 6.29 percentage points. This indicates that ESCA material possesses superior pore-filling capacity for STS. In addition to hydration reactions, ion exchange processes also occur during stabilization, and the hydration products intertwine with ionic compound crystals to form a spatial network structure that encapsulates the soil particles, thereby enhancing the overall strength of the stabilized soil. Similar microstructural densification has also been reported in stabilized soils incorporating GGBS, FA, FGDG, or other supplementary cementitious materials, where the formation of AFt, C-S-H, and C-A-S-H gels contributes to particle bonding and pore refinement [36,37]. In this study, the denser hydration products and lower pore area ratio observed in the ESCA-stabilized specimen further confirm that the multi-component ESCA system is more effective in improving the microstructure of STS than P.O 42.5.

### 3.6. CT Analysis

**Figure 12:**
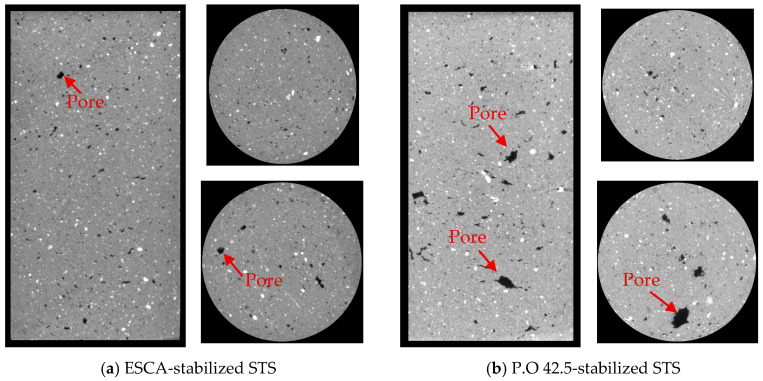
2D images of STS specimens stabilized with the two stabilizers. (**a**) ESCA-stabilized STS specimen; (**b**) P.O 42.5-stabilized STS specimen.

**Figure 13:**
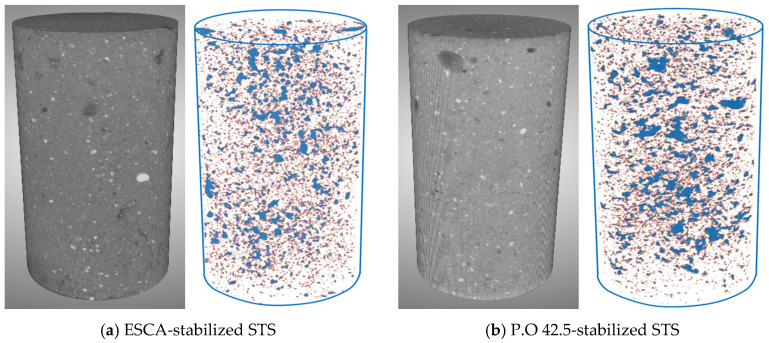
3D overall structure and pore CT images of STS specimens stabilized with the two stabilizers. (**a**) ESCA-stabilized STS specimen; (**b**) P.O 42.5-stabilized STS specimen.

**Figure 14:**
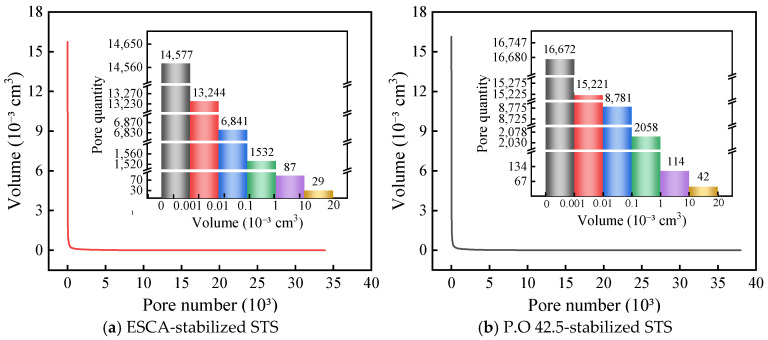
Ranking results and volume distribution interval statistics of internal pores in (**a**) ESCA-stabilized and (**b**) P.O 42.5-stabilized STS specimens.

As a non-destructive testing technique, industrial CT scanning can effectively characterize the internal microstructure of materials, thereby revealing the evolution mechanisms of their macroscopic mechanical properties. CT scanning was performed on two groups of STS specimens stabilized with ESCA and P.O 42.5 at a dosage of 20%. Typical 2D CT images of the specimen side and cross-section were obtained, and the results are presented in Figure 12. The 2D CT images were reconstructed into 3D models, and the internal pores of the specimens were extracted using the threshold segmentation method. The overall 3D spatial distribution of the specimens and their internal pores is shown in Figure 13. The pores in Figure 13 were further sorted in descending order of volume, and the volume distribution intervals and quantities of the pores were statistically analyzed, with the results presented in Figure 14. As observed in Figure 12, Figure 13 and Figure 14, at the same stabilizer dosage, the internal pores of the P.O 42.5-stabilized specimen were more developed than those of the ESCA-stabilized specimen, with obvious large pores visible in the 2D CT images. Meanwhile, quantitative analysis results indicated that the number of pores in the P.O 42.5-stabilized specimen was higher than that in the ESCA-stabilized specimen across all pore volume intervals. This difference is mainly related to the superior fluidity and cementitious activity of ESCA, which facilitates particle coating, pore filling, and interparticle bonding. These microstructural features provide direct evidence for the higher strength of ESCA-stabilized specimens. In addition, the pores in the ESCA-stabilized specimen were mostly concentrated near the specimen surface, which is primarily caused by the adhesion effect of the mold wall on the slurry during specimen preparation. In contrast, the P.O 42.5-stabilized specimen contained numerous large-sized pores distributed throughout its interior, which weakened the overall load-bearing capacity of the specimen and adversely affected its strength. From an engineering perspective, the reduced internal porosity of ESCA-stabilized STS is beneficial for improving structural compactness, reducing potential weak zones, and enhancing the reliability of solidified spoil used as recycled geomaterials. This is particularly important for the rapid treatment and resource utilization of shield tunnel spoil in urban underground construction.

## 4. Conclusions

In this study, the fundamental properties of ESCA and P.O 42.5 were comparatively analyzed, and the macroscopic mechanical behavior and microscopic solidification mechanisms of STS solidified with the two stabilizers were systematically investigated. The main conclusions are as follows:

(1) ESCA exhibits good fluidity even at low w/s ratios and has significantly shorter setting times than P.O 42.5. Moreover, its compressive strength at curing ages of 3, 7, and 28 days is markedly higher than that of P.O 42.5, demonstrating excellent early strength and rapid setting characteristics.

(2) The solidification efficiency of ESCA for STS is superior to that of P.O 42.5. Within the dosage range of 10% to 50%, the peak strength, elastic modulus, mass, and P-wave velocity of ESCA-stabilized specimens are all higher than those of P.O 42.5-stabilized specimens. Specifically, the peak strength of ESCA-stabilized specimens ranges from 1.09 to 1.40 times that of P.O 42.5-stabilized specimens, and the elastic modulus ranges from 1.26 to 2.16 times that of the latter. At a low dosage of 10%, the strength of ESCA-stabilized specimens can reach over 2 MPa, demonstrating excellent solidification capability at low stabilizer contents.

(3) Owing to its superior fluidity, ESCA can more fully penetrate and uniformly coat STS particles during mixing, forming tighter interparticle cementation and significantly reducing the internal porosity of the specimens, thereby enhancing the overall compactness and strength of the solidified soil. Microscopic analysis further confirms that the hydration products in the ESCA solidification system intertwine to form a spatial network structure, which is the fundamental reason for the improvement in macroscopic performance.

## Figures and Tables

**Table 1:** Basic physical properties of the STS.

Parameter	Value
Density *ρ*_d_/(g/cm^3^)	2.52
Water content *w*/%	23.12
Liquid limit *w*_L_/%	27.34
Plastic limit *w*_P_/%	18.95
Plasticity index *I*_P_	8.39
pH value	7.16
Optimum moisture content *w*_op_/%	22.41
Maximum dry density *ρ*_dmax_/(g·cm^−3^)	1.83

## Data Availability

The original contributions presented in this study are included in the article. Further inquiries can be directed to the corresponding author.
